# Transactions of the Massachusetts Homœopathic Medical Society

**Published:** 1890-02

**Authors:** 


### Transactions of the Massachusetts Homœopathic Medical Society.

We have received Vol. XI of this publication. It contains several practical articles worthy of perusal.

